# Effects of Incorporating Dry Matter Intake and Residual Feed Intake into a Selection Index for Dairy Cattle Using Deterministic Modeling

**DOI:** 10.3390/ani11041157

**Published:** 2021-04-17

**Authors:** Kerry Houlahan, Flavio S. Schenkel, Dagnachew Hailemariam, Jan Lassen, Morten Kargo, John B. Cole, Erin E. Connor, Silvia Wegmann, Oliveira Junior, Filippo Miglior, Allison Fleming, Tatiane C.S. Chud, Christine F. Baes

**Affiliations:** 1 Centre for Genetic Improvement of Livestock, Department of Animal Biosciences, University of Guelph, Guelph, ON N1G 2W1, Canada; khoulaha@uoguelph.ca (K.H.); schenkel@uoguelph.ca (F.S.S.); gerson@uoguelph.ca (O.J.); fmiglior@uoguelph.ca (F.M.); tchud@uoguelph.ca (T.C.S.C.); 2 Department of Agricultural, Food and Nutritional Science, University of Alberta, Edmonton, AB T6G 2P5, Canada; hailemar@ualberta.ca; 3 Viking Genetics, Ebeltoftvej 16, 8960 Assentoft, Denmark; JaLas@VikingGenetics.com; 4 Center for Quantitative Genetics and Genomics, Faculty of Science and Technology, Aarhus University, 8830 Tjele, Denmark; morten.kargo@mbg.au.dk; 5 SEGES, Agro Food Park 15, 8200 Aarhus N, Denmark; 6 Henry A. Wallace Beltsville Research Centre, Animal Genomics and Improvement Laboratory, USDA Agricultural Research Service, Beltsville, MD 20705, USA; john.b.cole@gmail.com; 7 Department of Animal and Food Sciences, University of Delaware, Newark, DE 19716, USA; eeconnor@udel.edu; 8 Qualitas AG, 6300 Zug, Switzerland; silvia_wegmann@bluewin.ch; 9 Lactanet Canada, Guelph, ON N1K 1E5, Canada; afleming@lactanet.ca; 10 Vetsuisse Faculty, Institute of Genetics, University of Bern, 3012 Bern, Switzerland

**Keywords:** feed efficiency, selection index, dairy cattle, residual feed intake

## Abstract

**Simple Summary:**

With the increasing cost of production, increasing global population, and a greater focus on sustainability, methods to improve cow efficiency are becoming critical for the dairy industry. An efficient cow is the one that produces the same amount of milk and milk solids while consuming less feed and remaining healthy and fertile; thus, allowing for a reduction of costs without reduced production. This simulation showed that directly selecting on feed conversion efficiency allowed for an economically advantageous and more balanced response to selection than indirect selection on feed intake. If too much selection pressure is placed on feed efficiency, there are negative implications for other traits within the selection index. Further work is required to optimize the methods for including feed efficiency in a selection index.

**Abstract:**

The inclusion of feed efficiency in the breeding goal for dairy cattle has been discussed for many years. The effects of incorporating feed efficiency into a selection index were assessed by indirect selection (dry matter intake) and direct selection (residual feed intake) using deterministic modeling. Both traits were investigated in three ways: (1) restricting the trait genetic gain to zero, (2) applying negative selection pressure, and (3) applying positive selection pressure. Changes in response to selection from economic and genetic gain perspectives were used to evaluate the impact of including feed efficiency with direct or indirect selection in an index. Improving feed efficiency through direct selection on residual feed intake was the best scenario analyzed, with the highest overall economic response including favorable responses to selection for production and feed efficiency. Over time, the response to selection is cumulative, with the potential for animals to reduce consumption by 0.16 kg to 2.7 kg of dry matter per day while maintaining production. As the selection pressure increased on residual feed intake, the response to selection for production, health, and fertility traits and body condition score became increasingly less favorable. This work provides insight into the potential long-term effects of selecting for feed efficiency as residual feed intake.

## 1. Introduction

With the global population rising rapidly, sustainable dairy production is a major research topic due to the demand for high-quality and sustainably produced dairy products [1]. Methods to increase the sustainability of the dairy industry are under continuous investigation. One potential way to improve on-farm efficiency is to breed for animals that are more feed efficient. Feed is a major expense for the dairy industry, accounting for over 50% of farm operations’ total costs [2,3]. Therefore, the efficiency with which cows convert feed to milk directly impacts farm costs and efficiency [4,5]. An efficient cow is the one that consumes less feed for the same amount of milk production while maintaining health and fertility; thus, allowing for a reduction of costs without reduced production [6].

Historically, genetic selection of dairy cattle was focused primarily on increasing milk production but additional traits, such as fertility and health, have been incorporated into selection programs around the world over the last 25 years [7,8]. Due to an increased focus on the financial aspects of dairy production, dairy producers prioritize selecting traits related to healthy and high-producing cows that are reproductively fit and have a long productive life in the herd. In Canada, there are currently two official selection indices: the Lifetime Performance Index (LPI) and Pro$, neither of which yet contain a direct feed efficiency (FE) trait [9,10]. Thus, there is an opportunity to include a FE trait in selection indices to improve the sustainability of Canadian dairy production.

There has been successful selection for FE in other livestock species, such as swine and poultry [11,12,13]. In dairy cattle, variation in the amount of feed consumed among animals of similar production levels has been observed, suggesting the ability to select for animals that are more efficient at feed utilization [14,15]. This observation has led to the investigation of including FE in breeding objectives worldwide [2,6,16,17,18]. The lack of practical methods for measuring feed intake on a large number of animals has hindered the incorporation of FE into breeding programs. Using technologies such as milk mid-infrared spectroscopy [19] and 3D cameras [20] to estimate feed intake could enable more large-scale phenotyping, allowing for easier implementation of FE into breeding programs [21,22]. With many aspects of including FE in breeding programs still unknown, the purpose of this research was to estimate preliminary genetic and phenotypic correlations for dry matter intake and residual feed intake with eight currently evaluated traits and to simulate the impact of including FE through direct and indirect selection in a selection index.

## 2. Materials and Methods

### 2.1. Data

#### 2.1.1. Trait Definitions

The evaluated traits were chosen as representatives for aspects of current selection that could be most impacted by the inclusion of novel traits and made up the core list of traits included in each scenario. The core traits were from first-lactation animals and included two production traits: fat yield (FY) and protein yield (PY); two fertility traits: age at first service for heifers (AFS) and interval from first service to conception for first parity cows (FSTC); two type traits: body condition score (BCS) and stature (STAT); and two health traits: clinical ketosis (CK) and displaced abomasum (DA). Fat yield and protein yield were measured as total 305 d yield in the first lactation. Age at first service was the difference between the date of birth and date of the first insemination, expressed in days. First service to conception was the number of days between the first service and successful conception in cows. Both health traits (CK and DA) were producer recorded and defined as binary traits, where 0 represented no incidence of the disease and 1 represented at least one case of the disease in the first lactation. The type traits (BCS and STAT) were assessed by trained classifiers at Holstein Canada (Brantford, ON, Canada) during the animals’ first lactation, with BCS being a measure of the fat covering over the tail head and rump on a scale of one (very thin) to five (very fat), and STAT was measured in centimeters from the top of the spine in between the hips to the ground. The novel traits selected for inclusion were dry matter intake (DMI) and, as a measure of FE, residual feed intake (RFI). Dry matter intake is a measure of feed intake and was defined as the average amount of dry matter (DM) consumed per day in kilograms by an animal for a standard 305 d lactation. Residual feed intake is a measure of FE that was defined as the difference between an animal’s expected feed intake based on requirements for maintenance and production and its actual feed intake [2,23]. To determine the effects of the novel traits on the core traits’ genetic trends and the impact on the overall selection index, selection pressure was placed on DMI and RFI separately.

#### 2.1.2. Data

The dataset for the eight core traits was provided by Lactanet Canada (Guelph, ON, Canada) and the dataset for DMI and RFI was provided by the Efficient Dairy Genome Project (https://www.genomedairy.ualberta.ca/, accessed on 15 October 15 2020). Data for DMI and RFI were available from five research stations located in Canada (University of Guelph, ON; University of Alberta, AB; Commercial Herd, Alberta), the United States of America (USDA-ARS, Beltsville, MD), Denmark (Aarhus University, Tjele), and Switzerland (Agroscope, Posieux). Data from a Canadian commercial herd was also available for this study. The data were standardized, and records were re-scaled to the mean and standard deviation for Canadian cows. Average daily DMI records were available for a 305 d lactation on 2,360 first-lactation animals. Residual feed intake was calculated for 2,030 animals as the residual of the linear fixed regression of DMI on energy corrected milk yield (ECM; [24]) and metabolic body weight (MBW; body weight^0.75^). The data on the eight core traits came from a random sampling of 5% of all Canadian herds in the database, apart from the three herds collecting DMI, ECM, MBW, and RFI. The resulting file contained around 150,000 animals. Further details on these data, including editing procedures and results for genetic and phenotypic correlations between the eight core traits, were presented by Oliveira et al. [25]. Trait definitions, genetic and phenotypic standard deviation, heritability, and breeding value accuracy are presented in Table 1.

### 2.2. Genetic Parameter Estimation

The genetic correlations among the eight core traits and the two novel traits were estimated with a series of bivariate animal models using WOMBAT version 07-02-2020 [28]. The effects considered in the models depended on the trait and followed Interbull recommendations for the eight core traits [25] and the model for DMI and RFI is presented below. The general statistical model was:

y=Xb+Za+Wh+e,

where: y is the vector of the observed phenotypes; b is the vector of fixed effects including year-season of calving (66 levels) and age at calving class (6 levels); a is the vector of random additive genetic effects; h is the vector of random herd-year of calving effects; X, Z, and ***W*** are incidence matrices relating b, a, and h with the phenotypic observations in y; and e is the vector of random residual error. The covariance matrices of a, h, and e were assumed to be:

$$var\left[\begin{array}{c} a\\ h\\ e \end{array}\right]=\left[\begin{array}{ccc} A\sigma_{a}^{2} & 0 & 0\\ 0 & I\sigma_{h}^{2} & 0\\ 0 & 0 & I\sigma_{e}^{2} \end{array}\right]$$

where $\sigma_{a}^{2}$ is the additive genetic variance, $\sigma_{h}^{2}$ is the herd-year variance, $\sigma_{e}^{2}$ is the residual variance, A is the numerator relationship matrix, and I is an identity matrix. The genetic parameters for the core traits are available in Table S1. The average genomic breeding value (GEBV) accuracy of 1390 bulls born between 2010 and 2018 was provided by Lactanet Canada for all core traits and used as an input parameter for the simulation together with the parameter estimates. The estimate of GEBV accuracy for DMI was provided by the Efficient Dairy Genome Project [26], while a literature estimate was used for the GEBV accuracy for RFI [27] as presented in Table 1.

### 2.3. Modeling Software

The modeling of including either DMI or RFI into a breeding program was done using ZPLAN+ (version 1.5.10, vit, Verden, Germany), a deterministic modeling program that can be used to model genetic and economic parameters within complex breeding programs [29]. The program utilizes selection index theory [30], the gene flow method [31], and economic modeling. To follow the gene flow, the population structure has to be defined using selection groups with their selection criteria, such as selection intensity and breeding goals. As already mentioned, an additional required input for the software is the accuracy of the GEBV.

### 2.4. Population Structure

**Figure 1:**
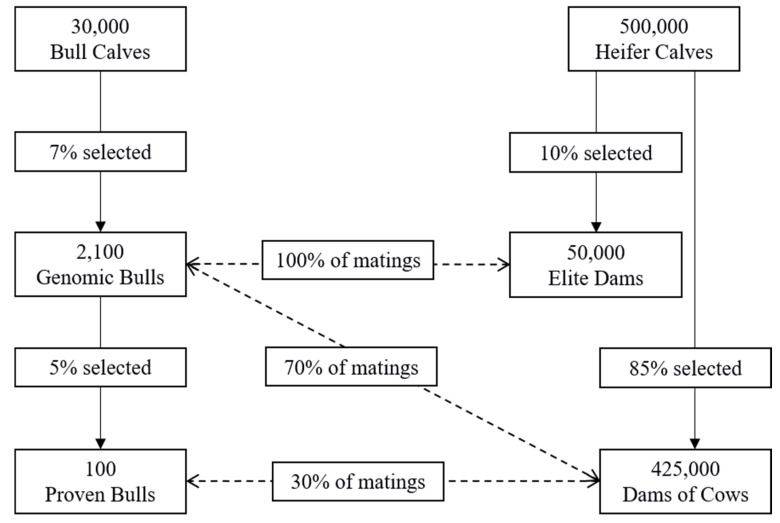
Diagram of the population breeding structure used in all scenarios.

This study focused on different scenarios with the same population structure. The male selection pathway contained three steps modeled to resemble the current Canadian setting. The simulation began with 30,000 genotyped bull calves (<1 year of age), where 2,100 (7%) became genomic bulls to be used for mating [32]. The genomic bulls, which are bulls for which breeding values are based solely on their genomic information, remained at this stage for three years, at which time 100 (5%) of these became proven bulls, which had 100 daughter records each [32]. Selected proven bulls remained as active breeding bulls for two additional years. The female selection pathway began with a population of 500,000 heifer calves, where 425,000 (85%) of the animals joined the milking herd at the time of first calving [33]. They remained in the herd as lactating cows for three lactations. From the initial population of 500,000 heifer calves, 50,000 (10%) became elite females, which were used to produce the next generation of bulls [33]. Elite females were mated exclusively to genomic bulls, while 70% of the general milking herd were mated to genomic bulls and 30% mated to proven bulls [34]. A visual representation of the population structure is presented in Figure 1. The allele flow matrix, where p_ij_ represents the proportion of alleles in class *i* at time *t* that come from class *j* at time *t*-1, for the breeding pathways is presented in Table 2. This matrix describes the source of all alleles in each age class [35].

### 2.5. Selection Scenarios

The incorporation of FE was carried out placing direct or indirect selection pressure on RFI, a measure of FE. To assess the impact of direct versus indirect selection, selection pressure was applied to DMI (indirect selection) or RFI (direct selection) using three methods: (1) restricting the trait genetic gain to zero (C); (2) applying negative (unfavorable) selection pressure (N); and (3) applying positive (favorable) selection pressure (P). For a baseline measurement, a scenario where DMI and RFI were included without selection pressure (BASE) was considered. Due to the high standard error for the estimated genetic correlations between the novel traits and currently recorded traits, two versions of each selection scenario were performed. The first version of each scenario (BASE, C, P, and N) used the estimated genetic parameters. The second version of each scenario (BASE_SD, C_SD, P_SD, and N_SD) used more conservative parameters, where the genetic parameters were adjusted by moving the correlation towards zero, either through adding or subtracting the respective standard error (Table S2). Index weights were optimized for each selection scenario, considering the economic value, genetic and phenotypic (co)variance matrices (Table S3).

A total of 14 scenarios were considered to assess the impact of including FE through indirect (DMI) or direct (RFI) selection pressure in a selection index. Selection scenarios are denoted by the novel trait under selection pressure (DMI or RFI), the scenarios utilizing the correlation corrected by the standard error are indicated by SD, and the type of selection for the novel trait. For example, DMI_N_SD indicates the scenario where DMI is included with a negative selection pressure, utilizing the adjusted correlation.

In addition to comparing the impact of selecting directly or indirectly on FE, the impact of increasing the positive selection pressure on RFI was explored. This was facilitated by doubling, tripling, quadrupling, and quintupling the economic value on RFI. Which was done because the economic values that were directly used to define the index weight in the selection index (see next section) would likely lead to a weak index weight for RFI. Scenarios were named following the same structure, where P denotes the positive selection pressure, with the addition of a number to indicate the multiplication factor of the economic value. For example, the scenario in which the RFI economic value was tripled was denoted by RFI_P3. Response to selection over time was calculated based on compound genetic gain and expressed in units of DMI per day.

### 2.6. Economic Values

Fat and protein yield are the main sources of revenue for producers in the Canadian dairy cattle industry [10]. Consequently, the assumed breeding objective was to improve FY and PY, while simultaneously improving health and fertility. Based on the trait definitions, improving health and fertility translates to a reduction of the incidence of disease and a reduction in the number of days at first service, and the number of days from the first service to conception. The economic values for conformation traits were optimized within each scenario to have no response to selection, in other words, to be held constant. The optimal economic value to hold the type traits constant was calculated using an Excel program [36]. All economic values presented are considered in Canadian dollars (CAD). Literature economic values in were: CAD 2.57 per day for AFS [37], CAD 6.86 per day for FSTC [38], CAD 233.00 per case of CK [39], and CAD 707.00 per case of DA [40]. Economic values (𝑣) were calculated for production traits by:

𝑣 = 𝑅 − 𝐶

𝐶 = *C1* ∗ *DMP1*

where 𝑣 is the economic value, 𝑅 is the revenue, 𝐶 is the cost, *C1* is the cost of 1 kg of DM, and *DMP1* is the amount of DM needed to produce 1.00 kg of the trait (fat or protein).

The cost of 1.00 kg of DM was assumed to be CAD 0.29 and the amount of DM needed to produce 1.00 kg of fat and protein was 6.00 kg and 3.70 kg, respectively [41]. Revenue for each trait was calculated by averaging the monthly producer-paid price in Canada for the trait from August 2019 to July 2020, which was CAD 10.76 and 8.23 per kg for fat and protein, respectively [42]. With that, the economic values for FY and PY were CAD 9.02 and 7.16, respectively. Conversely, health and fertility traits were assigned negative economic values, as the breeding goal was to reduce the incidence of disease, age at first service, and the number of days between first service and conception traits.

Each selection strategy for incorporating DMI or RFI had an economic value to achieve the selection goal of the specific index. The economic values for both DMI and RFI were the cost of DM associated with a 1 kg/day change in efficiency over a 305 d lactation. When holding either DMI or RFI constant in the C scenarios, an Excel program [36] was used to determine the optimal economic value. Given the above assumptions, when the breeding goal was to place positive selection pressure on the trait, a positive economic value (88.45) was placed on DMI and a negative economic value (−88.45) was placed on RFI. The inverse was true when placing negative selection pressure on the trait. When increasing positive selection pressure on RFI, the economic value was doubled (−176.90, RFI_P2), tripled (−265.35, RFI_P3), quadrupled (−353.80, RFI_P4), and quintupled (−442.25, RFI_P5).

## 3. Results

### 3.1. Genetic Parameters

This work provided preliminary estimates for the relationship of DMI and RFI with currently evaluated traits (Table 3). The data available for animals who had records for DMI and RFI along with all other recorded traits were limited; therefore, some parameter estimates had large standard errors (from 0.09 to 0.23 and 0.14 to 0.29 for genetic correlations and 0.02 to 0.17 and 0.02 to 0.21 for phenotypic correlations, for DMI and RFI, respectively).

Favorable genetic correlations were found between both production traits with DMI (0.43 for FY and 0.50 for PY). Production traits had genetic correlations with RFI less than 0.10. The genetic correlations (standard deviation) between DMI and health or fertility ranged from weak −0.13 (0.16) for CK to strong −0.61 (0.17) for AFS. Similar patterns were observed for the genetic correlations between RFI and health or fertility, ranging from −0.04 (0.29) for FSTC to −0.41 (0.24) for AFS. Strong genetic and phenotypic correlations of 0.68 (0.07) and 0.82 (0.02), respectively, were observed between DMI and RFI.

### 3.2. Direct vs. Indirect Selection on Feed Efficiency

#### 3.2.1. Index Response

The economic index response is presented in Table 4. The most favorable scenario from an economic perspective is selecting directly on FE with positive selection pressure on RFI (RFI_P). The index RFI_P was CAD 5.14 more profitable compared to the BASE. In all cases, scenarios containing selection pressure on DMI were not as profitable as the BASE, with a difference of up to CAD 45.33. The other two scenarios involving selection pressure on RFI (RFI_C and RFI_N) were also less profitable than the BASE, with a difference of CAD 9.88. The most economically favorable scenario in this study was RFI_P.

#### 3.2.2. Trait Response

To assess the individual trait response when selecting on FE, trait response for all traits were standardized using the genetic standard deviation for the respective trait (Table 4). The most favorable response to selection for RFI (−0.15 standard deviation units (SDU)) was observed in DMI_C; however, DMI_C also had the least favorable response to selection for FY, PY, BCS, AFS, FSTC, CK, and DA. Compared to BASE, there was an unfavorable response to selection in DMI_C with a difference of 0.07 SDU for FY and FSTC, 0.08 SDU for DA 0.11 SDU for PY and BCS, and 0.20 SDU for AFS. While DMI_N and RFI_P had similar responses to selection for all traits, including FE (−0.02 SDU), economically RFI_P was more profitable. Response to selection for FY and PY was similar between scenarios, except for DMI_C. This trend was also observed for AFS, FSTC, CK, DA, and DMI. The trait most affected by including FE directly into the selection index (RFI_P), was BCS where there was an unfavorable change of 0.08 SDU from the BASE.

### 3.3. Response of Feed Efficiency over Time

**Figure 2:**
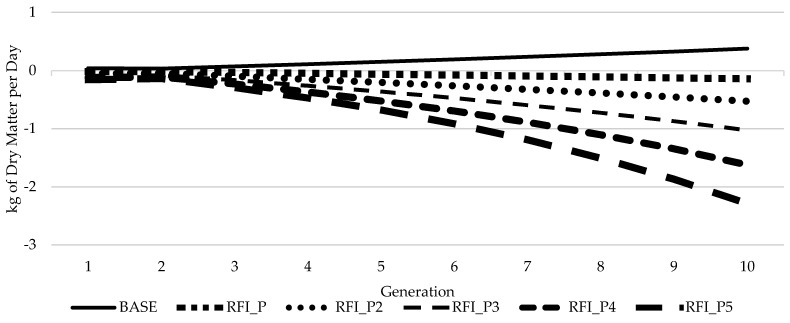
Cumulative response to selection for dry matter intake per day based on different weights of selection pressure on RFI over 10 years.

**Figure 3:**
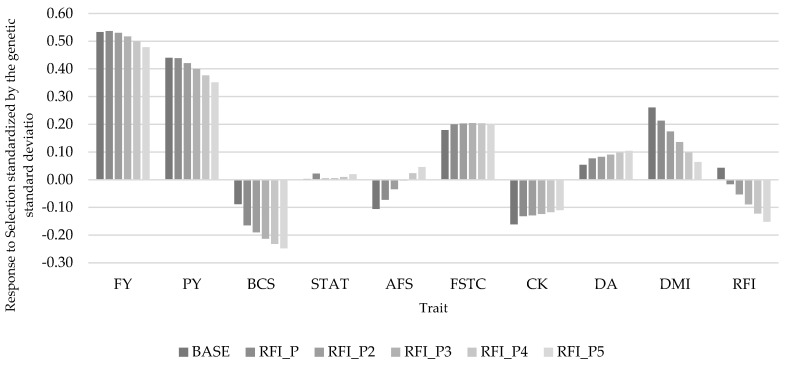
Response to selection for scenarios with different weights on RFI by trait standardized by the genetic standard deviation.

Considering FE over time based on this simulation, if FE is not selected for within a breeding program (BASE scenario) there is the potential for animals to become less efficient with cows eating an additional 0.43 kg of DM/day, or 131 kg of DM/305 d lactation after 10 generations. Placing selection pressure (RFI_P) on FE has the potential to improve FE with cows eating 0.16 kg of DM/day or 49.0 kg of DM/305 d lactation less after 10 generations, compared to the BASE scenario. If a greater selection pressure were placed on RFI to improve FE (e.g., 5 times more than in scenario RFI_P), the potential improvement could reach 2.77 kg of DM/day or 845 kg of DM/305 d lactation compared to not selecting on FE after 10 generations (Figure 2). When selection pressure is increased on RFI, there was an unfavorable change in the response to selection on the other traits within the selection index (Figure 3). Most notably, unfavorable changes in BCS and AFS of 0.16 SDU for each trait were observed between RFI_P and RFI_P5.

## 4. Discussion

### 4.1. Genetic Parameters

The heritability estimates (Table 1) for DMI (0.23 ± 0.04) were in line with previously reported estimates [43,44,45]. Additionally, the heritability for RFI (0.13 ± 0.03) was in agreement with previous studies that used similar RFI calculation equations [5,46,47]. These estimates indicate that these two traits are moderately heritable and, therefore, are reliable candidates for a selection program. The favorable genetic correlations of the production traits with DMI and RFI were in agreement with previously reported estimates [48,49]. Low phenotypic correlations between production traits and RFI are expected, as RFI is the residual term from a model containing production traits. The non-significant, low to moderate, and unfavorable correlations between RFI and the health and fertility traits suggest that health and fertility traits could have an undesired response to selection for FE if they are not considered in the selection index. The strong genetic and phenotypic correlations between DMI and RFI were slightly higher than reported by Lin et al. [50] and Manafiazar et al. [51]; however, still in the same positive direction. Overall, given the high standard error, genetic and phenotypic correlations with DMI and RFI require further investigation using a larger population.

### 4.2. Response to Selection of Feed Efficiency

Including FE in a selection index through direct or indirect selection has previously been investigated by Kennedy et al. [11] and Lu et al. [52]. The results of this study agreed with their conclusions, where it was possible to achieve a similar response to selection on FE using direct and indirect selection. Direct selection on FE occurs when the selection pressure is placed directly on the trait, RFI for this study, and indirect selection for FE occurs when the components of FE are included in the selection index. The components of FE are traditionally considered to be feed intake, milk production, and body maintenance [21,22]. In this study, the traits that were considered as the components of FE are DMI, FY, and PY (production) and BCS and STAT (body maintenance). Other traits that could replace those used in this study are energy-corrected milk and metabolic body weight, which would represent the production and body maintenance components, respectively [21,22,45]. It is important to note that FE in this study is a residual trait (RFI), meaning it is the residual of a linear regression of DMI on the energy sink traits. Seymour et al. [53] suggested that challenges are surrounding a residual trait for FE, as the model factors considered impact the estimation of RFI. In this study, the R^2^ of the model to calculate RFI was 0.27, which highlights the point that including additional information in the regression model could impact the residual term. With this information in mind, this study showed that it was more economically advantageous to directly select on FE as opposed to indirect selection using DMI.

While the maximum response to selection for FE was observed when DMI was held constant (DMI_C), it came at a great cost for the response to selection for all other traits. This was highlighted by the economic response of DMI_C, which had the lowest economic response of all the scenarios. This observation was in line with previous studies that suggested a limitation of DMI can lead to a limitation on milk synthesis and other biological processes [53,54]. The unfavorable response for all other core traits in the index, and corresponding poor economic index response, lead to the conclusion that selection directly on RFI is the best option for FE improvement. To further solidify that selecting directly on RFI was the best method for improving FE, RFI_P was the most profitable scenario in this study. It is important to note that the economic responses to the indices presented here are not representative of the true economic response in the industry but rather provide a method to compare the impact and assess the best methodology for selection to improve FE. 

The trait that was most unfavorably impacted by including FE directly into the selection index was BCS, which was expected as RFI and BCS had a moderately positive genetic correlation in this study. Because BCS can be considered an energy sink and included in the calculation of RFI, it has been proposed to include BCS in the regression model on DMI [53]. By including BCS in the regression model, RFI and BCS become phenotypically independent of each other which has the potential to help mitigate the unfavorable correlated response. Another way to mitigate any negative impacts could be to consider the lactation in multiple stages, where FE is only selected for after-peak lactation, avoiding the stage of lactation where animals are in a negative energy balance (NEB) [55]. Negative energy balance is a period where cows use more energy than they can consume, which requires the mobilization of body stores to meet energy demands [56]. When body stores are mobilized, appetite decreases further while milk production is climbing [57]. This leads to animals appearing to be very efficient in early lactation, when in fact, this is a fabrication of being in NEB. Applying selection pressure to animals based on apparent efficiency in early lactation would further exacerbate the issues that come with being in NEB. Additionally, cows with a high BCS pre-calving mobilize more body reserves post-calving, further reducing feed intake while producing even more milk in early lactation [58]. Due to the influence of BCS and impact of NEB in early lactation, focusing selection after peak lactation, even though a genetic correlation would likely exist between stages of lactation, would provide a more accurate picture of FE and has fewer potentially negative impacts on other traits.

Long-term selection on FE will result in animals that are better able to convert feed to milk. Australia successfully implemented a FE trait known as Feed Saved into their selection index [6]. Feed Saved is a combination of a genomic RFI component and an EBV from body weight predicted from type traits [6]. They have seen success with their breeding program, where animals one standard deviation above the mean consume 65 kg less feed per year while maintaining the same level of milk production [59]. More recently, the United States of America has also included Feed Saved into their selection program, where Feed Saved is expressed as the expected pounds of feed saved per lactation based on body weight composite and RFI [60]. The exact weighting that should be placed on FE for optimal progress was not analyzed in this study. Instead, we increased the weighting on FE to observe the impact on other traits. As the weight on RFI increases, the response to selection for RFI increases; however, the response to selection for all other traits considered becomes more unfavorable. In the long term, placing direct selection pressure on FE is the best way to ensure genetic progress.

## 5. Conclusions

This study simulated the impacts of direct and indirect selection on FE. The best scenario to improve FE in this study was to place direct selection pressure on RFI. To ensure the validity of this study, it should be replicated with a larger dataset for variance components estimation of the novel traits. The scenario in which selection pressure was placed directly on FE was the best economically, and there was a minimal unfavorable impact on most of the other traits within the index. Based on this study, over time the cumulative response to selection for RFI should lead to improvement of FE with the potential for animals to be 0.52 kg DM/day or 158 kg DM/305 d lactation more efficient than not selecting for FE after 10 generations.

## Figures and Tables

**Table 1:** Trait definitions, genetic and phenotypic standard deviations, heritability estimates, and genomic accuracy.

Trait	Definition	Genetic Standard Deviation	Phenotypic Standard Deviation	Heritability	GEBV Accuracy
FY	Fat yield (kg) during a 305 d lactation	30.67 ^1^	70.13 ^1^	0.29 ^a,1^	0.80
PY	Protein yield (kg) during a 305 d lactation	21.33 ^1^	55.45 ^1^	0.22 ^a,1^	0.79
BCS	The measure of the fat covering over the tail head and rump on a scale of 1 (very thin) to 5 (very fat) in first lactation	0.15 ^1^	0.35 ^1^	0.23 ^a,1^	0.77
STAT	Measure (cm) from the top of the spine in between hips to ground in first lactation	2.19 ^1^	4.42 ^1^	0.47 ^a,1^	0.77
AFS	Number of days from birth to first insemination	10.91 ^1^	56.08 ^1^	0.04 ^a,1^	0.69
FSTC	Number of days from first service to conception in first lactation	7.45 ^1^	44.68 ^1^	0.03 ^a,1^	0.74
CK	Binary scored trait (0:no case/unknown, 1:at least one case) in first lactation	0.03 ^1^	0.21 ^1^	0.02 ^a,1^	0.61
DA	Binary scored trait (0:no case/unknown, 1:at least one case) in first lactation	0.03 ^1^	0.15 ^1^	0.04 ^a,1^	0.59
DMI	Average dry matter intake per day for a 305 d lactation	1.54	3.25	0.23 ^b^	0.59 ^2^
RFI	Average residual feed intake per day for a 305 d lactation	0.89	2.50	0.13 ^b^	0.40 ^3^

^a^ standard deviation less than 0.01, ^b^ standard deviation between 0.01 and 0.05, ^1^ Oliveira Jr. et al. [25], ^2^ Miglior et al. [26], ^3^ Pryce et al. [27]. GEBV = genomic breeding value, FY = fat yield, PY = protein yield, BCS = body condition score, STAT = stature, AFS = age at first service, FSTC = first service to conception, CK = clinical ketosis, DA = displaced abomasum, DMI = dry matter intake, RFI = residual feed intake.

**Table 2:** The allele flow matrix ^1^ showing selection groups, where bulls refer to all breeding males and cows refer to all breeding females.

-Sex	Bulls						Cows				
	-Time	1	2	3	4	5	1	2	3	4	5
Bulls	1	0	0.167	0.167	0.167	0	0	0.125	0.125	0.125	0.125
	2	1	0	0	0	0	0	0	0	0	0
	3	0	1	0	0	0	0	0	0	0	0
	4	0	0	1	0	0	0	0	0	0	0
	5	0	0	0	1	0	0	0	0	0	0
Cows	1	0	0.117	0.117	0.192	0.075	0	0.125	0.125	0.125	0.125
	2	0	0	0	0	0	1	0	0	0	0
	3	0	0	0	0	0	0	1	0	0	0
	4	0	0	0	0	0	0	0	1	0	0
	5	0	0	0	0	0	0	0	0	1	0

^1^ The allele flow matrix, where *p_ij_* represents the proportion of alleles in class *i* at time *t* that come from class *j* at time t-1. This matrix describes the source of all alleles in each age class [35].

**Table 3:** Additive genetic and phenotypic correlation estimates (±standard errors).

Trait-	-	FY	PY	BCS	STAT	AFS	FSTC	CK	DA
DMI	$\rho_{g}$	0.43 ± 0.09	0.50 ± 0.09	0.14 ± 0.14	0.05 ± 0.13	−0.61 ± 0.17	−0.13 ± 0.23	−0.07 ± 0.21	−0.13 ± 0.16
	$\rho_{p}$	0.29 ± 0.02	0.29 ± 0.02	0.01 ± 0.02	0.25 ± 0.04	−0.05 ± 0.04	0.04 ± 0.04	0.23 ± 0.14	0.15 ± 0.17
RFI	$\rho_{g}$	−0.07 ± 0.14	0.08 ± 0.14	0.35 ± 0.17	−0.16 ± 0.15	−0.41 ± 0.24	−0.04 ± 0.29	−0.09 ± 0.26	−0.19 ± 0.21
	$\rho_{p}$	0.03 ± 0.03	0.03 ± 0.02	0.03 ± 0.02	0.08 ± 0.04	−0.05 ± 0.04	0.05 ± 0.04	−0.32 ± 0.16	−0.31 ± 0.21

FY = fat yield (kg), PY = protein yield (kg), BCS = body condition score (score), STAT = stature (cm), AFS = age at first service (days), FSTC = first service to conception (days), CK = clinical ketosis (case), DA = displaced abomasum (case), DMI = dry matter intake (kg), RFI = residual feed intake (kg), $\rho_{g}$ = genetic correlation, $\rho_{p}$ = phenotypic correlation.

**Table 4:** Genetic gain per year (in genetic standard deviations) and the total index response to selection (CAD).

Scenario	FY (kg)	PY (kg)	BCS (Score)	STAT (cm)	AFS (Days)	FSTC (Days)	CK (Case)	DA (Case)	DMI (kg)	RFI (kg)	Total Index Response (CAD)
BASE	0.53	0.44	−0.09	0.00	−0.11	0.18	−0.16	0.05	0.26	0.04	206.44
BASE_SD	0.53	0.44	−0.08	0.00	−0.10	0.18	−0.16	0.06	0.21	0.02	204.45
DMI_C	0.46	0.33	−0.20	0.00	0.09	0.25	−0.13	0.13	0.00	−0.15	161.11
DMI_P	0.53	0.43	−0.05	0.00	−0.11	0.17	−0.18	0.05	0.27	0.06	167.16
DMI_N	0.54	0.42	−0.11	0.00	−0.04	0.21	−0.18	0.08	0.19	−0.02	174.05
DMI_C_SD	0.44	0.28	0.05	0.00	0.02	0.11	−0.29	0.04	0.00	−0.09	167.12
DMI_P_SD	0.51	0.44	−0.08	0.00	−0.14	0.18	−0.13	0.05	0.25	0.06	167.05
DMI_N_SD	0.53	0.41	−0.11	0.00	−0.05	0.19	−0.18	0.07	0.13	−0.03	183.04
RFI_C	0.53	0.41	−0.05	0.01	−0.07	0.17	−0.21	0.06	0.22	0.00	196.57
RFI_P	0.54	0.44	−0.16	0.02	−0.07	0.20	−0.13	0.08	0.21	−0.02	211.58
RFI_N	0.51	0.43	−0.02	0.00	−0.14	0.16	−0.18	0.03	0.30	0.10	194.81
RFI_C_SD	0.51	0.41	0.00	0.00	−0.10	0.15	−0.21	0.04	0.18	0.00	200.15
RFI_P_SD	0.53	0.44	−0.15	0.00	−0.08	0.19	−0.13	0.07	0.17	−0.02	211.19
RFI_N_SD	0.52	0.43	−0.05	0.01	−0.12	0.19	−0.17	0.05	0.23	0.06	196.89

FY = fat yield (kg), PY = protein yield (kg), BCS = body condition score (score), STAT = stature (cm), AFS = age at first service (days), FSTC = first service to conception (days), CK = clinical ketosis (case), DA = displaced abomasum (case), DMI = dry matter intake (kg), RFI = residual feed intake (kg), C = trait held constant, P = positive (favorable) selection pressure, N = negative (unfavorable) selection pressure.

## Data Availability

This study was based on simulated data and all information needed to replicate the simulation process is provided in the material and methods.

## Supplementary Materials

The following are available online at https://www.mdpi.com/article/10.3390/ani11041157/s1, Table S1: Genetic (above) and phenotypic (below) correlations used for DMI and RFI scenarios, Table S2: Genetic (above) and phenotypic (below) correlations used for DMI_SD and RFI_SD scenarios, Table S3: Optimized index weights by trait for the BASE, DMI and RFI scenarios.
