# Supplementary material for: Effects of Incorporating Dry Matter Intake and Residual Feed Intake into a Selection Index for Dairy Cattle Using Deterministic Modeling
Source: Animals (Basel). 2021 Apr 17;11(4):1157. doi: 10.3390/ani11041157 (PMC8072614; doi:10.3390/ani11041157)
Supplement: Supplementary file 1 [file animals-11-01157-s001.zip › Supplementary Table 2-Houlahan et al.pdf]

# SUPPLEMENTARY MATERIAL Houlahan et al.

**Supplementary Table 2**. Genetic (above) and phenotypic (below) correlations used for DMI_SD and RFI_SD scenarios

| | FY | PY | BCS | STAT | AFS | FSTC | CK | DA | DMI | RFI |
|---|---|---|---|---|---|---|---|---|---|---|
| FY | | 0.64[1] | -0.23[1] | 0.05[1] | -0.15[1] | 0.30[1] | 0.30[1] | 0.13[1] | 0.34 | 0.07 |
| PY | 0.79[1] | | -0.21[1] | 0.03[1] | -0.20[1] | 0.37[1] | 0.15[1] | 0.13[1] | 0.41 | -0.07 |
| BCS | -0.08[1] | -0.07[1] | | 0.07[1] | -0.02[1] | -0.03[1] | -0.05[1] | -0.05[1] | 0.00 | 0.18 |
| STAT | 0.07[1] | 0.11[1] | -0.04[1] | | -0.10[1] | 0.02[1] | 0.00[1] | 0.03[1] | -0.08 | -0.01 |
| AFS | 0.00[1] | -0.01[1] | -0.12[1] | -0.45[1] | | 0.08[1] | 0.01[1] | 0.00[1] | -0.44 | -0.17 |
| FSTC | 0.15[1] | 0.18[1] | -0.25[1] | 0.12[1] | -0.02[1] | | 0.02[1] | 0.05[1] | 0.10 | 0.26 |
| CK | 0.01[1] | -0.01[1] | -0.56[1] | 0.05[1] | -0.08[1] | 0.35[1] | | 0.21[1] | 0.14 | 0.17 |
| DA | -0.03[1] | -0.04[1] | -0.30[1] | 0.24[1] | -0.02[1] | 0.22[1] | 0.61[1] | | 0.04 | 0.02 |
| DMI | 0.26 | 0.26 | -0.02 | 0.22 | -0.01 | 0.00 | 0.09 | -0.02 | | |
| RFI | 0.01 | 0.01 | 0.01 | 0.04 | -0.01 | 0.01 | -0.16 | -0.10 | | |

[1]Oliveira Jr. et al., 2021; FY = fat yield (kg), PY = protein yield (kg), BCS = body condition score (score), STAT = stature (cm), AFS = age at first service (days), FSTC = first service to conception, CK = clinical ketosis (case), DA = displaced abomasum (case), DMI = dry matter intake (kg/day), RFI = residual feed intake (kg/day)
