# Supplementary material for: Effects of Incorporating Dry Matter Intake and Residual Feed Intake into a Selection Index for Dairy Cattle Using Deterministic Modeling
Source: Animals (Basel). 2021 Apr 17;11(4):1157. doi: 10.3390/ani11041157 (PMC8072614; doi:10.3390/ani11041157)
Supplement: Supplementary file 1 [file animals-11-01157-s001.zip › Supplementary Table 3-Houlahan et al.pdf]

SUPPLEMENTARY MATERIAL Houlahan et al.

Supplementary Table 3. Optimized index weights by trait for BASE, DMI and RFI scenarios.

	FY	PY	BCS	STAT	AFS	FSTC	CK	DA	DMI	RFI
BASE	1.62	0.93	-174.16	-5.49	-0.08	-0.26	-2322.96	-4986.05	-	-
BASE_SD	1.62	0.93	-174.16	-5.49	-0.08	-0.26	-2322.96	-4986.05	-	-
DMI_C	1.60	0.90	-467.57	-1.28	-0.05	-0.26	-2565.29	-5361.86	78.93	-
DMI_P	1.67	0.97	972.00	-14.27	-0.10	-0.30	-1440.32	-3210.25	90.97	-
DMI_N	1.64	0.94	474.70	-9.20	-0.07	-0.29	-1848.97	-3932.65	70.64	-
DMI_SD_C	1.74	1.01	3296.95	-27.01	-0.07	-0.39	197.50	505.86	-51.36	-
DMI_SD_P	1.63	0.94	-112.66	-5.72	-0.10	-0.26	-2278.73	-4902.71	24.79	-
DMI_SD_N	1.61	0.92	-360.10	-4.16	-0.07	-0.26	-2466.01	-5258.72	-14.50	-
RFI_C	1.71	1.00	1987.47	-20.08	-0.08	-0.35	-457.51	-808.34	-	-28.06
RFI_P	1.61	0.92	-360.10	-4.16	-0.07	-0.26	-2466.01	-5258.72	-	-14.50
RFI_N	1.69	0.99	1622.19	-14.79	-0.10	-0.30	-1050.41	-2761.74	-	-140.94
RFI_SD_C	1.73	1.02	2662.75	-23.70	-0.09	-0.37	-183.43	-315.81	-	-10.57
RFI_SD_P	1.59	0.91	-1248.20	1.95	-0.07	-0.23	-3017.04	-6455.01	-	-204.54
RFI_SD_N	1.65	0.96	713.69	-10.04	-0.09	-0.28	-1755.89	-3837.34	-	-96.30

FY = fat yield (kg), PY = protein yield (kg), BCS = body condition score (score), STAT = stature (cm), AFS = age at first service (days), FSTC = first service to conception, CK = clinical ketosis (case), DA = displaced abomasum (case), DMI = dry matter intake (kg/day), RFI = residual feed intake (kg/day), C = trait held constant, P = positive (favorable) selection pressure, N = negative (unfavorable) selection pressure
